# Relationships between foot type and dynamic rearfoot frontal plane motion

**DOI:** 10.1186/1757-1146-3-9

**Published:** 2010-06-16

**Authors:** Vivienne H Chuter

**Affiliations:** 1 Discipline of Podiatry, Faculty of Health, University of Newcastle, Ourimbah, New South Wales, Australia

## Abstract

**Background:**

The Foot Posture Index (FPI) provides an easily applicable, validated method for quantifying static foot posture. However there is limited evidence relating to the ability of the FPI to predict dynamic foot function. This study aimed to assess the relationship between dynamic rearfoot motion and FPI scores in pronated and normal foot types.

**Methods:**

40 participants were recruited with equal numbers of pronated and normal foot types as classified by their FPI score. Three dimensional rearfoot motion was collected for each of the participants. Dynamic maximum rearfoot eversion was correlated with the total FPI score across all participants and within the normal and pronated foot types. Linear correlations were performed between components of the total FPI scores measuring frontal plane rearfoot position and maximum rearfoot eversion. The capacity of the total FPI score to predict maximum frontal plane motion of the rearfoot was investigated using linear regression analysis.

**Results:**

The correlation between the total FPI score and maximum rearfoot eversion was strongly positive (r = 0.92, p < 0.05). Correlation performed on data subsets demonstrated the pronated foot type (FPI = +6 to +9) and maximum rearfoot eversion angle were more strongly positively correlated (r = 0.81, p < 0.05) than the normal foot type (FPI = 0 to +5) and maximum rearfoot eversion (r = 0.76, p < 0.05). Correlations between frontal plane rearfoot FPI score and frontal plane motion during gait were strongly positive, (r = 0.79 p < 0.05 pronated group, r = 0.71 p < 0.05 normal group), however were less strong than the total FPI score and rearfoot motion. Linear regression analysis demonstrated a significant and strong relationship between the total FPI score and maximum rearfoot eversion (r^2 ^= 0.85, p < 0.001).

**Conclusions:**

The results of this study suggest the FPI has strong predictive ability for dynamic rearfoot function. This will assist in clinical screening and research by allowing easy classification by functional foot type. Positive correlations between frontal plane rearfoot measurements and maximum rearfoot eversion suggest the FPI may identify dominant planar components of dynamic rearfoot motion and warrants further investigation.

## Background

Foot posture has been implicated in biomechanical dysfunction of the lower limb and a variety of overuse injuries [1-3]. Many static measures have been developed to describe foot posture and subsequently investigated as possible predictors of dynamic rearfoot motion [4,5]. Measures have included frontal plane calcaneal angle, (frequently referred to as rearfoot angle), medial arch angle and arch height, however, none has consistently been found to be accurate predictors of dynamic rearfoot motion for stance phase [4-8]. The clinical and research benefits of having an easily performed static measurement capable of predicting dynamic function are significant, potentially assisting in improved accuracy of clinical screening and orthotic prescription, and standardisation of functional foot type for research.

The six item Foot Posture Index, (FPI), uses a validated criterion-based observational measurement of the forefoot and rearfoot in a static position [9]. The reference system differs from previously described classification systems due to the number of observations recorded, the inclusion of multi-segment and multiplanar measurements evaluating foot position on a continuum relative to pes planus or cavus position and the ease of application of the model.

Measurement of the rearfoot includes a combination of transverse and frontal plane assessments including talar head palpation, curvature above and below the malleolus and frontal plane position of the calcaneus. The forefoot measurements combine transverse and sagittal plane measurements including prominence of a talonavicular bulge, forefoot transverse plane position and sagittal plane congruence of the medial longitudinal arch. A score is allocated to each measure to give a total overall score indicative of foot posture with reference values provided for classification purposes [9].

Previous research assessing the capacity of the FPI to predict dynamic function has assessed three dimensional inversion/eversion of the ankle joint complex during the midstance of walking and midfoot motion measured via video gait analysis and electromagnetic motion tracking. Results so far have indicated a weak relationship between the static FPI measurement and dynamic foot function [9,10]. Electromagnetic tracking of the ankle joint complex in a small group of participants demonstrated the FPI predicted 41% of variance in ankle joint complex inversion and eversion [9]. The study involved FPI being manipulated through use of inverted or everted wedging and the resulting ankle joint complex gait dymanics being correlated to the contrived FPI during midstance. Whilst this demonstrates relatively poor predictive capacity, it is of greater strength than similar investigations of alternative static measures [5,11]. In relation to the midfoot, 45% of variance in minimal navicular height and 13.2% variance in navicular drop were found to be predicted by the FPI suggesting poor prediction of forefoot motion however, this is restricted to motion measured with two dimensional techniques [10].

Due to the limited number of studies investigating the use of the FPI as a predictor of dynamic function the results are inconclusive. The purpose of this study was to determine and compare the strength of correlation between static foot position, as determined by the FPI, and maximum dynamic three dimensional frontal plane rearfoot eversion in both pronated and normal foot types. Overall predictive ability of the total FPI score for dynamic rearfoot motion was investigated.

Planar dominance of subtalar joint motion has been linked to subtalar joint axis position, specifically the pitch of the axis, with increased frontal plane motion of the rearfoot thought to be associated with a lower pitched axis [12]. The correlation between the score for the rearfoot frontal plane components of the FPI measurement and pure frontal plane motion of the calcaneus was calculated to determine the strength of relationship between static frontal plane dominance at the subtalar joint and dynamic frontal plane motion.

## Methods

This project was undertaken in the Biomechanics Department of the School of Exercise and Sports Science, Faculty of Health Sciences, Cumberland Campus of the University of Sydney. Ethical approval was obtained from the University of Sydney's Ethics Committee. Informed written consent was given by all participants prior to their participation in this study.

### Participants

Twenty male and 20 female participants were recruited from the University of Sydney student population for participation in this study, mean age 32.4 yrs (SD ± 4.7 yrs), mean height 171 cm (SD ± 8.9 cm) and mean weight 69.5 kg (SD ± 4.1 kg). Only data for the right foot was included. Participants were classified as either pronated or normal according to reference values provided for the FPI with a normal foot classified with a score of 0 to +5 and +6 to +9 indicative of a pronated foot type. Equal numbers of males and females and pronated and normal foot types were recruited into each group.

### Procedure

FPI was determined for all participants recruited for this study by an experienced clinician. Inclusion criteria for the study required a pronated or neutral foot type as determined by the total FPI score when applied by an experienced clinician. Participants who had a negative FPI score indicating a pes cavus foot type were excluded from the study. Participants with history of major lower limb or back trauma, surgery or any systemic disorder affecting the musculoskeletal system were excluded from the study.

**Figure 1:**
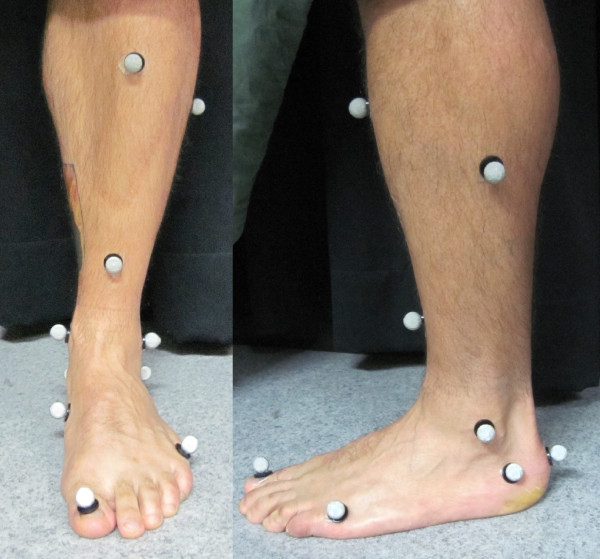
**Frontal and sagittal plane views of the marker set used for the definition of segments**.

Three dimensional motion of an 11 point retro-reflective marker set attached to the subject's right limb was collected using a Motion Analysis 9-video camera system (Falcon 8 mm, Motion Analysis Corp., Santa Rosa, CA) and a motion analysis system EvaRT 3.4 (Motion Analysis Corp.). Markers were applied to the hallux, head of the fifth metatarsal and navicular for the forefoot segment. The rearfoot and shank consisted of medial, lateral and posterior calcaneal markers and medial and lateral malleolar and upper, lower and lateral tibial makers (Figure 1). Leg markers were 1 cm in diameter, foot markers ranged from 0.5 cm-0.75 cm in diameter. The marker set was used to create a rigid three-segment, three dimensional lower limb model consisting of forefoot, rearfoot, and shank [4]. The cameras were arranged around a central 15 m walkway, creating a capture volume approximately 2.5 m long, 1.5 m high and 1 m wide, varying slightly according to the height and leg length of the subject. Kinematic data were collected at 120 Hz.

Participants were required to perform barefoot walking trials. A reference trial with the subject standing in the anatomical position at natural angle and base of gait was taken prior to the walking trials. The participants were instructed to walk through the capture area. Walking trials were collected at a speed of 1.4 m/s. Trials falling more than 10% outside these velocities were excluded. A minimum of five acceptable walking trials were performed by each subject as this has been shown to provide consistent kinematic data [13].

**Figure 2:**
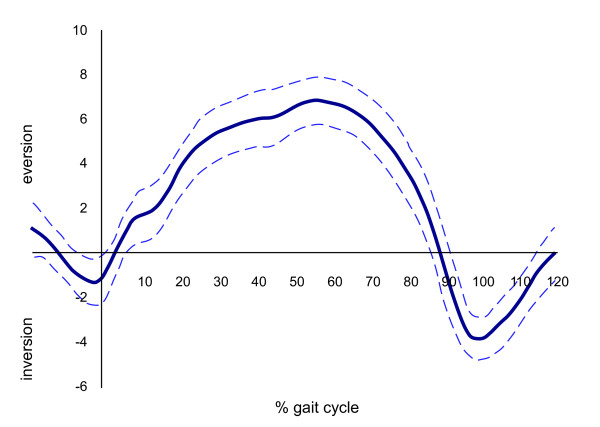
**Walking gait frontal plane rearfoot motion mean (N = 1, FPI Score +6) with 95% confidence intervals**.

Kinematic data were low pass filtered at 6 Hz using a zero phase second order Butterworth filter. Three dimensional marker position coordinates were processed using Kintrak 6.3, (The University of Calgary, Calgary, Canada) to obtain joint angular displacement of the rearfoot relative to the shank. Trials were normalised to 120% of stance (including 20% prior to heel strike) and kinematic data were then processed using a MatLab program (The Maths Works Inc., MA) to determine the discrete variable (maximum eversion) to be entered into the statistical analysis. Figure 2 demonstrates a typical rearfoot frontal plane motion time series output.

### Statistical analysis

Ordinal FPI data were converted to Rasch transformed scores allowing the data to be analysed as interval data [14]. Linear correlations were performed to identify the strength of relationship between maximum dynamic rearfoot eversion and the total FPI score within the entire population and within pronated and normal groups. A possible relationship between evidence of frontal plane dominance of the subtalar joint, and maximum rearfoot eversion and was also examined [12]. Planar dominance was determined via a breakdown of individual scores for the FPI. Subject scores relating to inversion and eversion of the calcaneus (associated with frontal plane motion) and curvature above and below the lateral malleolus (representing a combination of frontal and transverse plane motion) were calculated and correlated with maximum measurements for eversion giving possible scores of -4 to +4 correlated against maximum angular eversion of the rearfoot [9].

Correlation values above 0.8 were considered very strong, between 0.6 and 0.8 strong and between 0.3 and 0.6 moderate. Correlation coefficient values below 0.3 were considered weak due to the relatively small sample size [15].

Data were assessed for normality of distribution via scatter plots and homogeneity of variance using Levene's test to determine suitability for linear regression analysis. Linear regression analysis was performed between the total FPI and maximum rearfoot eversion to determine predictive capacity of rearfoot motion for the total FPI score. All statistical analysis was performed using SPSS version 17 (SPSS Science, Chicago, Illinois) software.

## Results

**Figure 3:**
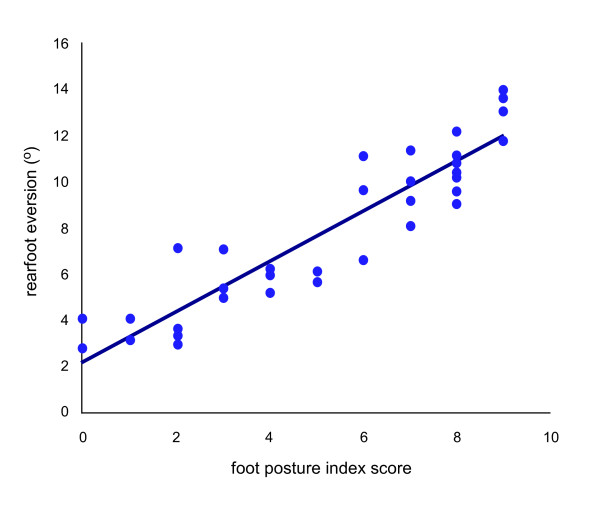
**Scatterplot maximum rearfoot eversion versus total FPI score, (r = 0.92, p < 0.05, n = 40)**.

Descriptive statistics relating to maximum rearfoot eversion angle are shown in Table 1. The total FPI score was correlated with maximum rearfoot eversion angle for the entire subject population (Figure 3). Positive correlation between the total FPI score and maximum eversion was found to be very strong (r = 0.92, p < 0.05) indicating close association between the total FPI score and maximum rearfoot eversion. Correlations between the FPI score and maximum rearfoot eversion angle were performed on data subsets representing a pronated foot group (FPI = +6 to +9) and a normal foot group (FPI = 0 to +5). The relationship between the FPI score and maximum rearfoot angle was stronger in the pronated group (r = 0.81, p < 0.05) than in the normal group (r = 0.76, p < 0.05).

**Table 1:** Descriptive Statistics: maximum rearfoot eversion angle

	N	Minimum (°)	Maximum(°)	Mean (°)	Std. Deviation (°)
Normal Group FPI = 0 to +5	20	3	7	4.95	1.16

Pronated Group FPI = +6 to +9	20	7	14	10.71	1.42

Correlations between frontal plane rearfoot FPI score and frontal plane motion during gait were strong and statistically significant across all participants (r = 0.83, p < 0.05), however, less strong than the total FPI score and rearfoot motion (r = 0.92), indicating the association between frontal plane score and maximum eversion angle is not as strong as the total FPI score and maximum rearfoot eversion angle. This was consistent with correlations of frontal plane rearfoot FPI score and frontal plane motion during gait within the pronated and normal groups which were strong (r = 0.79, p < 0.05, pronated group, r = 0.71, p < 0.05 normal group), however, were less strong than the relationship between the total FPI and maximum reafoot eversion (0.81, p < 0.05 and 0.76, p < 0.05 for the pronated and normal groups respectively).

Linear regression analysis demonstrated a significant and strong relationship between the total FPI score and maximum rearfoot eversion (r^2 ^= 0.85, p < 0.001) for the entire subject cohort (n = 40). Therefore, the total FPI score can be considered to be highly predictive of maximum rearfoot eversion angle across normal and pronated foot types.

## Discussion

Correlations of the total FPI score and maximum rearfoot eversion angle for both the pronated and normal foot types demonstrated a significant positive relationship (r = 0.81 and r = 0.76 respectively). Linear regression analysis suggests strong predictive capacity of the FPI for frontal plane motion of the rearfoot (r^2 ^= 0.85, p < 0.001) with the FPI predicting 85% of the variation in maximum eversion angle. This is in contrast to initial investigations of the relationship between FPI and dynamic foot function which demonstrate a weaker relationship between both dynamic midfoot and ankle joint complex motion and static FPI scores [9,10]. One previous study evaluated ankle joint complex motion and the FPI score in manipulated positions [9]. The method of measuring maximum rearfoot eversion in unmodified gait and in a larger sample may explain the increased strength of relationship found in this study. Furthermore, in this study FPI scores were correlated with maximum rearfoot eversion whenever this occurred during stance phase allowing for an inter-relationship between the midfoot and forefoot to be included. This allowed for delayed or prolonged rearfoot eversion, both recently identified as distinct patterns of rearfoot motion [16] to be included in the statistical tests.

Investigation of the relationship between the FPI frontal plane score of the rearfoot and maximum eversion angle demonstrated a strong, statistically significant relationship between the two variables for both the pronated foot type group and the normal foot type group. The pronated group demonstrated the stronger correlation with rearfoot motion, most likely due to greater range of pronation providing measureable differences in the individual planar components of rearfoot pronation. The presence of a positive relationship in a relatively small cohort suggests that further investigations are required, particularly relating to a highly pronated foot type (FPI 10+) which is more likely to demonstrate significant differences across the three planes of motion making up subtalar pronation. Correct identification of dominant planar components of rearfoot motion may potentially assist with orthotic prescription, specifically in relation to the position of the point of correction and the style of the device, with frontal plane dominance suggesting increased calcaneal motion control is required.

Modern three-dimensional motion analysis techniques used for collection of rearfoot data from participants in this study may also have contributed to findings of much stronger predictive ability of the FPI than in results for midfoot dynamic motion captured with Video Sequence Analysis as published previously [10]. Similarly, isolation of this study to the rearfoot ensured movement from multiple joints in the midfoot were not included. The ability of a static postural measurement to predict dynamic midfoot function may be reduced as movement occurs across multiple joints simultaneously with individual axes of motion. The midfoot FPI measurements also concentrate on medially located structures, (talo-navicular congruence and medial arch height) however, during gait movement occurs across the entire midfoot.

There are several limitations to this study that should be considered. This study was restricted to normal and pronated foot types as determined by FPI score. A supinated foot type, classified by a score -5 to 0 on the FPI scale, was not included. Due to the nature of the ordinal scale used in the FPI, i.e. evenly distributed categories and directional, it suggests that the predictive capacity of the FPI may extend to a negatively scored supinated foot type however this is currently an assumption.

In this study the investigation of the effect of planar dominance, (identified by a breakdown of the FPI scores), assumed the measurement of curvature above and below the lateral malleolus to be a frontal plane measurement. In reality, the FPI scoring system identifies this as a combination of frontal and transverse plane position [9]. Therefore, this study potentially overestimates the strength of the relationship between dynamic frontal plane motion of the rearfoot and frontal plane dominance in the FPI score.

Analysis was restricted to the frontal plane due to frontal plane motion of the rearfoot being adequately demonstrated by calcaneal motion allowing comparison between static measurements and dynamic function. Components of the FPI related to the static transverse plane position (assessed by palpation of the talar head) were not compared to dynamic motion as talar head motion cannot be accurately or reliably measured by skin mounted markers. There is no component of sagittal plane position included in the rearfoot FPI scoring system therefore this could not be included.

## Conclusions

The FPI is a validated, quick and simple clinical measurement which can be easily applied. The findings of this study suggest that it may be an important and convenient screening tool in evaluation of foot function and subsequent predisposition to injury.

Historically, research into the effect of foot orthoses and footwear on dynamic foot function has been hampered by difficulty in reliably classifying foot type for inclusion in studies, possibly contributing to subject-specific findings and lack of homogenous response to specific orthotic styles [17,18]. The results of this study suggest that the FPI has a strong positive relationship with maximum eversion of the rearfoot and is capable of predicting 85% of the variance in maximum eversion during the stance phase of gait. This suggests the FPI has significant predictive ability for dynamic rearfoot function which may assist in clinical screening and in the future research of the effect of orthotic prescription on foot function in specific cohorts.

Positive correlations between frontal plane rearfoot measurements and maximum rearfoot eversion suggests the FPI may also have a role in identifying dominant planar components of dynamic rearfoot motion and warrants further investigation.

## Competing interests

The author declares that they have no competing interests.
